# Identifying Potential Norovirus Epidemics in China via Internet Surveillance

**DOI:** 10.2196/jmir.7855

**Published:** 2017-08-08

**Authors:** Kui Liu, Sichao Huang, Zi-Ping Miao, Bin Chen, Tao Jiang, Gaofeng Cai, Zhenggang Jiang, Yongdi Chen, Zhengting Wang, Hua Gu, Chengliang Chai, Jianmin Jiang

**Affiliations:** ^1^ Zhejiang Provincial Center for Disease Control and Prevention Hangzhou China; ^2^ Key Laboratory of Vaccine, Prevention and Control of Infectious Disease of Zhejiang Province Hangzhou China; ^3^ School of Medicine, Ningbo University Ningbo China

**Keywords:** norovirus, Internet surveillance, disease prediction

## Abstract

**Background:**

Norovirus is a common virus that causes acute gastroenteritis worldwide, but a monitoring system for norovirus is unavailable in China.

**Objective:**

We aimed to identify norovirus epidemics through Internet surveillance and construct an appropriate model to predict potential norovirus infections.

**Methods:**

The norovirus-related data of a selected outbreak in Jiaxing Municipality, Zhejiang Province of China, in 2014 were collected from immediate epidemiological investigation, and the Internet search volume, as indicated by the Baidu Index, was acquired from the Baidu search engine. All correlated search keywords in relation to norovirus were captured, screened, and composited to establish the composite Baidu Index at different time lags by Spearman rank correlation. The optimal model was chosen and possibly predicted maps in Zhejiang Province were presented by ArcGIS software.

**Results:**

The combination of two vital keywords at a time lag of 1 day was ultimately identified as optimal (ρ=.924, *P*<.001). The exponential curve model was constructed to fit the trend of this epidemic, suggesting that a one-unit increase in the mean composite Baidu Index contributed to an increase of norovirus infections by 2.15 times during the outbreak. In addition to Jiaxing Municipality, Hangzhou Municipality might have had some potential epidemics in the study time from the predicted model.

**Conclusions:**

Although there are limitations with early warning and unavoidable biases, Internet surveillance may be still useful for the monitoring of norovirus epidemics when a monitoring system is unavailable.

## Introduction

Acute gastroenteritis, inflammation of the gastrointestinal tract, is defined as the sudden onset of diarrhea, with or without signs of nausea, vomiting, fever, or abdominal pain [1,2]. The known pathogenic causes of acute gastroenteritis include various infective pathogens and other noninfectious causes. Infectious acute gastroenteritis is generally caused by enteric viruses, bacteria, and protozoal pathogens. Norovirus, a single-stranded RNA virus of the *Caliciviridae* family, is a leading cause of infectious acute gastroenteritis worldwide across all age groups, particularly in health care and community settings [3,4]. In the United States, norovirus causes nearly 21 million cases of acute gastroenteritis annually, and nearly 50% of acute gastroenteritis occurrences across Europe were attributed to the norovirus infection [5,6]. Although norovirus infection is viewed as a self-limited illness, it might be still responsible for severe dehydration, and even potential death, in children and the elderly population [7-9]. Infection might be attributable to closed bedside care and exposure to vomit of contaminated food, water and aerosol of norovirus, and other factors such as prolonged time for viral shedding and the enhanced viability in the environment might play vital roles in improving transmissibility of norovirus [4,10-12]. Additionally, no obvious evidence supports the idea of there being a specific reservoir, and only the scattered speculation from available literature viewed immunocompromised individuals, elderly, and malnourished hosts as potential norovirus reservoirs [13]. Available studies from surveillance systems demonstrate that 0.7% of reported outbreaks were foodborne, 28.5% were person-to-person, and the remaining 70.8% were unclear or not described [10]. Transmission of norovirus commonly occurs before the typical symptoms appear, which further increases the difficulty for interventions [11]. Thus, how to effectively supervise and control norovirus infection has aroused substantial concern around the world.

Norovirus was ranked the second most common etiological agent only after rotavirus in children younger than 5 years in China [14-16]. Field surveys imply that unboiled water and contaminated food are the common causes of norovirus infection [12,17]. The high contagiosity, frequent virus mutation, and limited immune protection has resulted in more frequent epidemics of norovirus outbreak on the Chinese mainland since the winter of 2014, especially in schools [18-20]. Norovirus infection is classified as “other infectious diarrhea” (excluding the illness of cholera, bacillary dysentery, amebic dysentery, and typhoid/paratyphoid) in the Chinese National Notifiable Infectious Disease Reporting System. Accordingly, norovirus cases are not reported in an independent module, and some norovirus cases manifesting the main symptom of vomiting are inevitably omitted as well. The Public Health Emergency Management Information System can only focus on some clustered epidemics; therefore, some sporadic cases or subclinical infections of norovirus may be omitted. Thus, effective interventions for norovirus at the early stage are a pressing issue for public health.

With the rapid development of Internet technology, an increasing number of researchers have tried to take advantage of the retrieval function of search engines to forecast and warn against infectious diseases [21,22]. In China, both wired and wireless networks have been booming to meet the increasing demands of the cyber citizens. The China Internet Development Statistical Report released on July 23, 2015, revealed a total of 668 million Internet users (48.8% of the population), 18.94 million more than 6 months previous [23]. As the most frequently used search engine, Baidu has a priority selection incidence of 89.1% among Chinese cyber users [24]. The Baidu Index, based on the search frequency of some keywords within the Baidu search engine, can be viewed as awareness and requirement of cyber users [25]. Evidence from previous studies of communicable diseases suggested a potential relationship between search volume and the number of infected cases [26,27]. One study also used the comprehensive Baidu Index to construct a linear regression model to predict the potential cases of epidemic erythromelalgia, suggesting that the Baidu Index may serve as a good early indicator for epidemics [22]. Given that there is no specific monitor system for norovirus in China, the purpose of this study was to determine whether Internet surveillance was a helpful supplement to traditional surveillance of norovirus epidemics in China.

## Methods

### Ethics

This study was approved by the Ethics Committee of Zhejiang Provincial Center for Disease Control and Prevention. Given no privacy information involving human participants, it was granted an exemption from informed consent.

### Information of Epidemic

**Figure 1:**
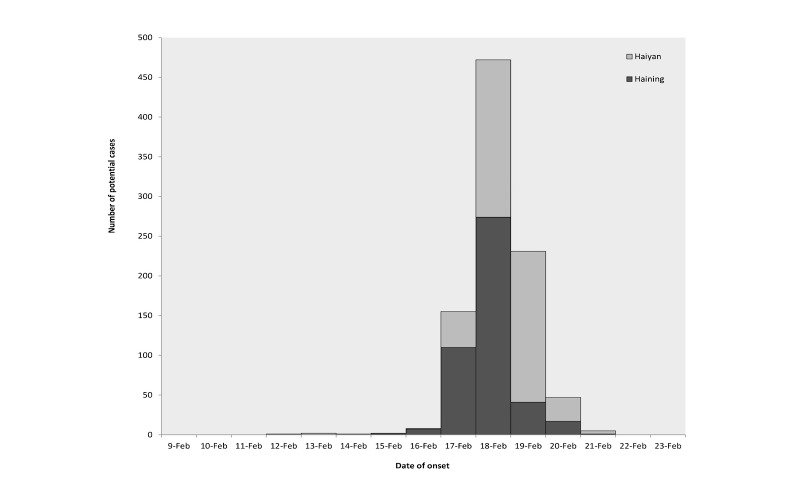
Daily cases of norovirus outbreak in Jiaxing Municipality from February 12 to 21, 2014, within the counties of Haiyan and Haining.

Clustered cases in schools of diarrhea and vomiting were notified in Haining and Haiyan, two counties in the Jiaxing Municipality, on February 17, 2014. After receiving notice, a field investigation conducted by the Zhejiang Provincial Center for Disease Control and Prevention was performed to find the potential causes. Using a standard questionnaire, potential cases were searched within seven schools in Haiyan and six schools in Haining. Through the field survey, the first case was identified on February 12, 2014, and the epidemic lasted for 10 days. The inclusion criteria of possible cases were vomiting more than once or having diarrhea more than three times in one day, including probable cases and confirmed cases. There were a total of 924 cases in this outbreak with a ratio of 1 male to 1.2 females (Figure 1).

### Baidu Index

The Baidu Index comprehensively reflects media exposure and users’ concerns based on certain keywords used by cyber users in the past day. Although the specific algorithm of Baidu Index is not available to the public because it is proprietary information, it was proven to be similar to Google Flu Trend in identifying public behavior on the Internet for different areas on diverse days [25]. Given the potential time lag between the onset of the symptoms in cases and related Internet searching, we collected the Baidu Index of Jiaxing Municipality from February 10 to 28, 2014. The data in the same period during 2013 and 2015 were also extracted.

### Keyword Screening and Data Collection

In China, the same idea can be expressed with diverse characters among different populations. That is to say, the retrieval of disease-related information may be distinctive through the search engine. Thus, how to recognize keywords specific to norovirus was vital for Internet surveillance. Furthermore, no standardized guidance was available for this issue; the disease names and main presentations had been commonly chosen as the primary keywords [21,22,28]. In this study, the primary keywords chosen (in Chinese) were “norovirus,” “nausea,” “emesis,” “abdominal pain,” and “diarrhea.” More norovirus-related keywords searched by cyber users were acquired at Keywords Mining, a website that uses semantic correlation analysis [29]. All keywords obtained from the website were stemmed from search engines and also websites, blogs, and other online sources.

We retrieved from this website the top 100 keywords for each of the five initial keywords. Two individuals evaluated these 500 keywords to exclude unrelated ones. In case of discrepancy in opinion, a third person made the final decision. Then, correlations between potential case number and Baidu Index of the screened keywords with possible time lags were calculated. If *P*<.05 combined with a Spearman rank correlation coefficient (ρ) >0.4, the keyword was brought into the group with the special time lag. Similar to a previous study, we chose the onset of illness as the beginning of the study time and had five groups with time lags of 0 to 4 days [22].

### Composite Baidu Index

**Figure 2:**
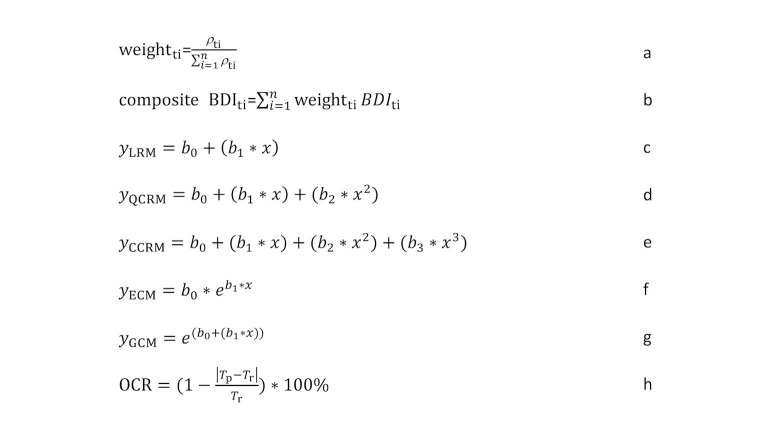
Equations used in the study.

After excluding keywords with no significance, the meaningful keywords were grouped by different time lags. In each group, the weight of each keyword (weight_ti_) and composite Baidu Index (composite BDI_ti_) were calculated as equation a and b in Figure 2, where *t* represents the potential time lag, *i* indicates the order number of present keyword, *n* is the number of keywords included in a specific time lag, *ρ*_ti_ is the Spearman rank correlation of included keyword (i) with specific time lag (t), and *BDI*_ti_ denotes the daily Baidu Index of included keywords (i) with specific time lag (t) [22].

### Optimal Time Lag and Model Construction

According to the Spearman rank correlation of the composite Baidu Index and potential case number in each time lag, the time lags with the superior coefficient were determined optimal. Additionally, the mean composite Baidu Index normalized by local netizens (1/100 million) was calculated to avoid potential bias stemming from the distinction of Internet users in diverse area (Multimedia Appendix 1). Prediction models were constructed to explore the relationship of the mean composite Baidu Index and potential case number under diverse optimal lag periods, respectively.

Given the distribution characteristics of potential norovirus cases, we constructed a linear regression model and latent curve models. In this study, five possible models shown in Figure 2 (c-g) were evaluated, including linear regression model (LRM), quadratic curve regression model (QCRM), cubic curve regression model (CCRM), exponential curve model (ECM), and growth curve model (GCM).

In these models, *b*_1_, *b*_2_, and *b*_3_ indicate the coefficients; *x* and *y* represent the mean composite Baidu Index and case number, respectively. The optimal model was examined by the *P* value of variance (ANOVA) and *t* test for coefficient. The overall coincidence rate (OCR) shown in Figure 2 (h) would be employed to determine the optimal model, in which *T*_p_ represents the total predicted case number from the specific prediction model during the study period, and *T*_r_ denotes the case number during the study period. The more the value of OCR trended to 100%, the better the selected model would be.

### Spatial Presentation for Predicted Norovirus

The composite Baidu Index of 11 municipalities in Zhejiang Province was calculated from February 10 to 28, 2014. Based on the optimal prediction model described previously, the predicted norovirus cases in 11 municipalities were acquired and shown by ArcGIS software. The predicted norovirus cases in the same period of 2013 and 2015 were also shown on the map.

### Statistical Analysis

All analyses were performed using SPSS Statistics 20.0 (SPSS Inc, Chicago, IL, USA). Spatial display was done with the Geographic Information System version 10.1 (SERI Inc, Redlands, CA, USA). Results were considered statistically significant if *P*<.05 with two sides.

## Results

### Epidemiological Characteristics of the Norovirus Outbreak

This epidemic was first reported in Haiyan, followed by Haining, within the Jiaxing Municipality in Zhejiang Province of China. A total of 924 cases (420 male and 504 female) involving 13 schools were detected from February 12 to 21, 2014; five were teachers and the rest were students. The clinical symptoms were mild and the main symptom was vomiting accompanied by nausea, diarrhea, fever, and abdominal pain, but no death occurred. The local departments of disease control in both counties responded rapidly to the epidemic, with such interventions as class suspension, disinfection within dormitories and classrooms, and sealing barreled water. Vomitus and anal swabs were retained from some cases. Given the potential exposure to barreled water among most cases, local centers for disease control sampled the different brands of barreled water in the schools. After 15:00 on February 21, no new cases were reported. Laboratory tests detected norovirus genogroup II in eight samples of anal swabs, five samples of opened barreled water, and one sample of unopened barreled water, suggesting that water contaminated by norovirus caused the epidemic. Through in-depth investigation of the drinking water, we eventually deemed the occurrences at two sites as one outbreak because the contaminated water was supplied by the same supplier. This detailed information had been described in another study [30].

### Optimal Time Lags of the Composite Baidu Index

Five possible time lags (0, 1, 2, 3, and 4 days) were considered to screen the considerable time lags, and details of all inclusion keywords for each time lag are listed in Table 1. The correlation coefficient peaked at the time lag of 2 days with five keywords (ρ=.945, *P*<.001). Considering the potential epidemiology significance and delicate difference at the time lag of 1 day with two keywords (ρ=.924, *P*<.001), both time lags were included to construct appropriate models.

### Prediction Model for Norovirus

**Figure 3:**
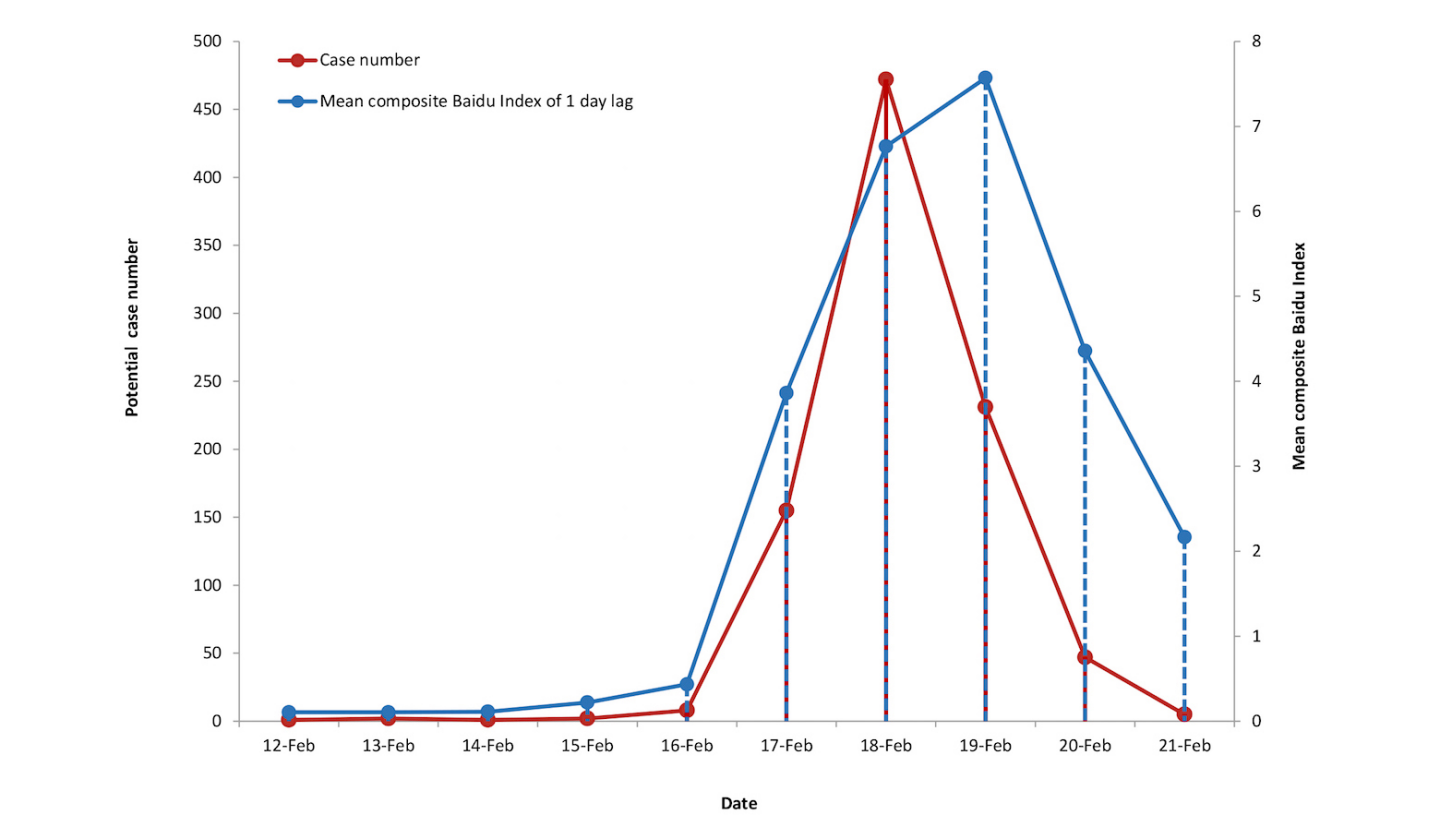
Fluctuant trend of potential case number and mean composite Baidu Index at the time lag of 1 day during February 12 to 21, 2014.

The composite Baidu Index was calculated with the weight and Baidu Index of the different keywords at lag times of 1 and 2 days, respectively. After the standardization of local netizens, the regression models were then constructed to predict the potential norovirus cases by the mean composite Baidu Index independent variable. Of the five candidate models considered in our study, ECM was determined the optimal model for the time lag of 1 day (Figure 3), whereas the top model for the time lag of 2 days was GCM (Table 2). Then, OCR values of both models in Jiaxing Municipality were calculated, demonstrating that OCR in ECM was 90.69% and in GCM was 66.00% (Table 3). Consequently, the optimal model was decided as ECM with 1 day lag. In this model, y=1.809*e^0.764*^^x^, which was interpreted as a one-unit increase in the mean composite Baidu Index contributed to an increase of norovirus infections by 2.15 times during the outbreak.

**Table 1:** Inclusive keywords at time lags of 0 to 4 days after screening.

Time lag and keyword		Indicators for keyword			Indicators for composition	
		ρ	*P*	Weight	ρ	*P*
**Day 0**					.740	.01
	Norovirus	.740	.01	0.348		
	Noro	.735	.02	0.346		
	Vomiting and bleeding	.650	.04	0.306		
**Day 1**					.924	<.001
	Noro	.950	<.001	0.507		
	Norovirus	.924	<.001	0.493		
**Day 2**					.945	<.001
	Norovirus	.945	<.001	0.237		
	Noro	.932	<.001	0.234		
	Vomiting and diarrhea	.715	.02	0.180		
	Nausea and vomiting	.701	.02	0.176		
	Viral diarrhea in infants	.688	.03	0.173		
**Day 3**					.707	.02
	Norovirus	.707	.02	0.266		
	Why feel headache and nausea	.665	.04	0.250		
	Noro	.648	.04	0.243		
	Why feel dizziness and nausea	.642	.045	0.241		
**Day 4**						
	Why feel headache and nausea	.673	.03	1	.673	.03

**Table 2:** Details of model screening for five potential candidate models.

Time lag and model		*F* (df1,df2)	*P*	*R*^2^	Coefficient							
					b_0_	*P*	b_1_	*P*	b_2_	*P*	b_3_	*P*
**Day 1**												
	LRM^a^	18.292 (1,8)	.003	.696	–22.213	.59	44.610	.003				
	QCRM^b^	9.254 (2,7)	.01	.726	–4.24	.93	10.957	.79	4.827	.41		
	CCRM^c^	7.901 (3,6)	.02	.798	30.292	.56	–151.010	.24	66.333	.17	–5.496	.19
	ECM^d^	59.664 (1,8)	<.001	.882	1.809	.03	0.764	<.001				
	GCM^e^	59.664 (1,8)	<.001	.882	0.593	.15	0.764	<.001				
**Day 2**												
	LRM	19.215 (1,8)	.002	.706	–35.840	.40	95.996	.002				
	QCRM	14.546 (2,7)	.003	.806	7.440	.86	–30.034	.68	36.277	.10		
	CCRM	9.450 (3,6)	.01	.825	–11.621	.82	103.430	.58	–67.747	.62	19.205	.45
	ECM	41.870 (1,8)	<.001	.840	1.535	.06	1.593	<.001				
	GCM	41.870 (1,8)	<.001	.840	0.428	.38	1.593	<.001				

^a^LRM: linear regression model.

^b^QCRM: quadratic curve regression model.

^c^CCRM: cubic curve regression model.

^d^ECM: exponential curve model.

^e^GCM: growth curve model.

**Table 3:** The overall coincidence rate (OCR) value of the exponential curve model (ECM) and growth curve model (GCM) with different time lags.

Indicators	ECM with 1 day time lag	GCM with 2 days time lag
Total predicted case number	1010	610
Case number during the study period	924	924
OCR	90.69%	66.00%

**Figure 4:**
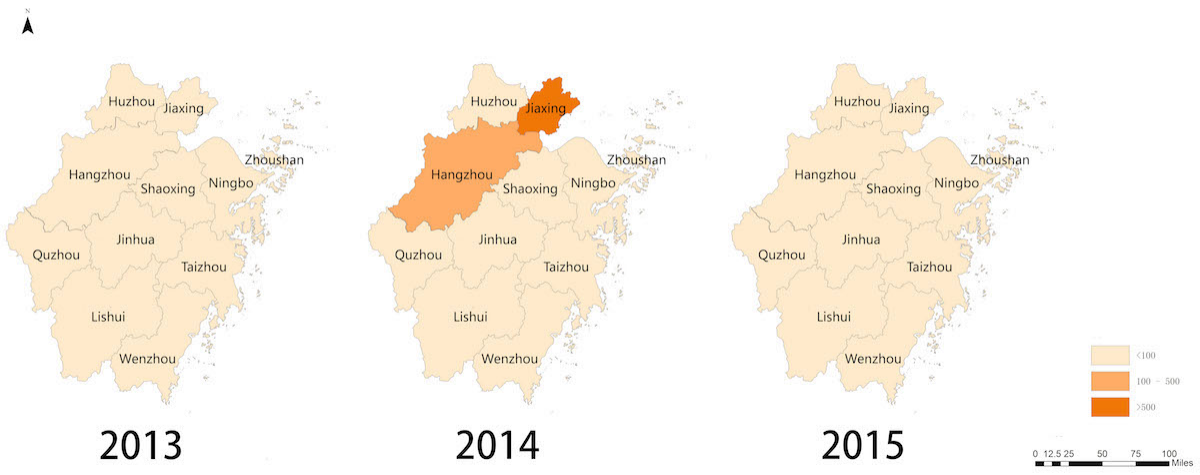
Predicted norovirus infections in Zhejiang Province from February 12 to 21 in each year from 2013 to 2015.

### Spatial Presentation for Predicted Norovirus

Based on the preceding optimal model, potential norovirus cases of 11 municipalities in Zhejiang Province during the study time were evaluated and displayed on the map. The number of possible cases in the same period in 2013 and 2015 was predicted (Figure 4). From the displayed map, Jiaxing Municipality in 2014 showed the peak of the norovirus infection than other areas in the same period. Moreover, there might have been potential norovirus epidemics in other municipalities, such as Hangzhou.

## Discussion

### Principal Findings

Studies demonstrated that norovirus, a common pathogen of acute gastroenteritis, caused several serious outbreaks in China especially in Zhejiang Province in the last decade, implying imminent demand for effective control and prevention of norovirus epidemics [31-34]. With an independent reporting module yet to be constructed in the Chinese National Notifiable Infectious Disease Reporting System, only norovirus outbreaks were recorded by the Public Health Emergency Management Information System. To some extent, such a circumstance limited the detection of norovirus epidemics at the early stage. Fortunately, Internet-based surveillance offers a potential means for monitoring emergent infectious diseases, whose effectiveness and dependability have been explored and examined in some studies [22,27,35].

In this study, we used Internet-based surveillance to identify the association between possible norovirus case number and the fluctuant retrieval index of norovirus-related keywords from the Baidu search engine, and explored an optimal model with specific time lag for the prediction of norovirus epidemics. Possible norovirus-related keywords were first captured at the Keywords Mining website, which involved the technology of text mining and semantic analysis. Then, the Spearman rank correlations between possible norovirus case numbers and the search index of norovirus-related keywords were calculated to determine the inclusion keywords with different time lags. After that, all inclusion keywords for each time lag were combined to obtain the composite Baidu Index and calculate its related Spearman rank correlation coefficients. In this research, the results suggested that the composite Baidu Index of five included keywords was significantly related to this outbreak at the time lag of 2 days with the largest Spearman rank correlation coefficient of ρ=.945 followed by two included keywords at the time lag of 1 day with Spearman rank correlation coefficient of ρ=.924. Combined with the OCR values of different models, ECM was shown to be optimal including two included keywords with a time lag of 1 day. These findings are similar to previous study implying Internet-based surveillance based on some specific keywords might be effective in identifying epidemics [22]. In contrast to a previous study, the optimal mean composite Baidu Index related to norovirus in this study was essentially on a parallel track with the new cases reported in Figure 3 [22]. Given the short disease course, the serious symptom of acute gastroenteritis, and the characteristics of self-healing, the clustered epidemics of norovirus infection in young groups were more likely to be monitored, particularly in schools. Thus, the early warning of norovirus epidemics by Internet surveillance might be limited for epidemics with short incubation periods and rapid disease progression. Moreover, according to ECM with a time lag of 1 day, the optimal model indicated that an increase of one unit in the composite Baidu Index from 1/100 million netizens contributed to the rise of norovirus infections by 2.15 times during the outbreak, which further supports the quantitative relationship between Internet surveillance and potential norovirus cases.

Previous studies have identified different optimal time lags in the analysis of diverse diseases, suggesting that this might be attributed to diverse study purposes, various incubation periods, and population susceptibility of different diseases [21,22,36]. Also, the ultimate time lag (1 day) selected in this paper was not the absolutely optimal time lag (2 days) in our study, implying more in-depth studies should be performed at more exquisite scope to explore its potential mechanism, which could mine significantly targeted interventions in public health.

Geographic information system technology has been adopted to present predicted norovirus cases in scale of Zhejiang Province (Figure 4). Compared with the same study period in each year from 2013 to 2015, our predicted results also demonstrated that the selected norovirus outbreak that occurred in Jiaxing Municipality was the largest one, which may prove the reliability of our prediction by Internet surveillance to some extent. Interestingly, some potential cases were identified in Hangzhou Municipality. Although no direct evidence was provided, Hangzhou was shown to be a high-risk region of norovirus infection in the available literature, which also certified the efficiency of Internet surveillance [37-39].

### Limitations

Some limitations should be mentioned in our study:

1. The representation of the study was limited because the norovirus outbreak in question involved only schools. Therefore, the conclusion extrapolated to the whole population was insufficient.
2. Although the clinical and epidemiological evidence could be obtained in this study, laboratory tests were not performed for all cases. Thus, the accuracy of the prediction might be affected.
3. The *R*^2^ of ECM was .882, whereas the rest (<12%) were not explored. Other external factors, such as environmental factors and economic factors, might influence the eventual results, which were not considered in this study.
4. Despite some technical means employed to search as many related keywords as possible, omission of subordinate keywords might be inevitable.
5. Other models that were not explored in this study might have a better goodness of fit, which could influence the accuracy of this study.

### Conclusions

Over the past decades, the development of the Internet and search engines in China have experienced rapid leaps. A majority of the public sought medical information and expressed personal concerns on the Internet, which provided the underlying possibility for disease surveillance through the Internet, particularly in the field of emergent infectious diseases. The role of forecasting and warning against infectious diseases through the Internet has been identified in some available studies, whereas there is still no record reporting acute infectious diseases such as norovirus that have a short incubation period. In this study, we try to explore the significant keywords involving norovirus, construct an effective model, and eventually identify the potential epidemics of norovirus in Zhejiang Province using Internet surveillance. Despite existing limitations in early warning and unavoidable biases, Internet surveillance may be still useful for the monitoring of norovirus epidemics when a monitoring system is unavailable.

## Appendix

Multimedia Appendix 1 The Internet users of different municipalities in Zhejiang Province by the end of 2013.

## Abbreviations

CCRM: cubic curve regression model
ECM: exponential curve model
GCM: growth curve model
LRM: linear regression model
QCRM: quadratic curve regression model
OCR: overall coincidence rate
