# Supplementary material for: Identifying Potential Norovirus Epidemics in China via Internet Surveillance
Source: J Med Internet Res. 2017 Aug 8;19(8):e282. doi: 10.2196/jmir.7855 (PMC5566627; doi:10.2196/jmir.7855)
Supplement: Multimedia Appendix 1 [file jmir_v19i8e282_app1.pdf]

The Internet users of different municipalities in Zhejiang Province by the end of 2013

Area	Hangzhou	Wenzhou	Ningbo	Jinhua	Taizhou	Jiaxing	Shaoxing	Huzhou	Lishui	Quzhou	Zhoushan
Internet users (1/100 million)	682	538	525	338	327	269	251	155	101	81	63
